# Dam (*Canis familiaris*) Welfare throughout the Peri-Parturient Period in Commercial Breeding Kennels

**DOI:** 10.3390/ani12202820

**Published:** 2022-10-18

**Authors:** Aynsley C. Romaniuk, Shanis Barnard, Jennifer E. Weller, Hsin-Yi Weng, Sriveny Dangoudoubiyam, Candace Croney

**Affiliations:** 1 Department of Comparative Pathobiology, Purdue University, 725 Harrison Street, West Lafayette, IN 47906, USA; 2 Animal Welfare Unit–Livestock Production Sciences Branch, Sustainable Agri-Food Sciences Division, Agri-Food and Biosciences Institute, Hillsborough BT26 6DR, UK

**Keywords:** dog, welfare, commercial dog breeding, cortisol, immunoglobulin A, behavior, peri-parturient period, gestation, lactation

## Abstract

**Simple Summary:**

Welfare problems experienced during gestation and lactation may negatively affect dams and their puppies. However, the welfare states of dams in commercial breeding (CB) kennels during this period have not been well examined. Therefore, we examined a range of behavioral, physical, and physiological metrics throughout the period around parturition to identify if changes indicative of impaired welfare were present. We tested 74 dams from eight CB kennels at 6 and 1 week prepartum, and 4 and 8 weeks postpartum. At each time point we measured their responses to a stranger approaching, their physical health, and indicators of their stress response, immune function, and parasite burden. Findings did not reveal major changes in dam welfare. Most changes observed were likely because of natural biological changes resulting from pregnancy, lactation, and weaning. However, as some changes in metrics deviated from what was expected and there were changes in environmental and management factors during this time, future research should identify how they affect dam welfare.

**Abstract:**

Poor dam welfare throughout the peri-parturient period can also negatively affect that of their offspring. This study aimed to identify changes in physical, physiological, and behavioral metrics indicative of dam welfare throughout the peri-parturient period. Dams (*n* = 74) from eight U.S. Midwest commercial breeding (CB) kennels were tested at 6 and 1 week prepartum, and 4 and 8 weeks postpartum. At each time point dams underwent a stranger approach test, physical health assessment, hair collection for hair cortisol concentration (HCC) and fecal collection for fecal glucocorticoid metabolites (FGM), fecal secretory immunoglobulin A (sIgA) and parasite detection. Linear mixed-effects models indicated dams exhibited more affiliative behaviors towards the stranger at 4 weeks postpartum than 6 weeks prepartum (*p* = 0.03), increased HCC from 4-weeks to 8 weeks postpartum (*p* = 0.02), and increased FGM from 1 week prepartum to 8 weeks postpartum (*p* = 0.04). At each respective time point, the percentage of dams with intestinal parasites was 11%, 4%, 23%, and 15%. Most changes are likely due to increased energy requirements and hormonal variations. However, deviations from expected changes may have resulted from changes in environment and/ or management, which should be explored in future studies.

## 1. Introduction

In altricial species, negative affective states such as fear are associated with prenatal stress and poor maternal care [1,2,3], which may negatively affect behavioral and physiological responses to acute stress in offspring (rats: [2,4,5,6,7]; blue foxes: [8]; rhesus monkeys: [9]). These associations between dam and offspring welfare have rarely been explored in dogs. Doing so is especially crucial for dogs originating from commercial breeding (CB) kennels where dams spend more time in the peri-parturient period than the average dog. They have approximately three litters within a 2 year period and this was the case for the dogs in this study. Furthermore, puppies from CB kennels are faced with the potential stressors of separation from their dams and litters, loss of familiar caretakers and environments, and transport before entering a new family home. CB kennels aid in supplying the demand for companion dogs in the U.S., however, public perceptions of them are typically negative. They are often reported by media to be squalid environments. However, direct observational studies have not found this to be the case for kennels in compliance with U.S. federal and state laws that determine minimum standards of care [10,11]. Instead, the majority of dogs in such kennels were reported to be physically and behaviorally healthy [12,13,14,15,16]. Before investigating the effects of maternal care and other external stressors on the development and welfare of puppies, it is important to accurately assess the dams’ welfare states throughout the peri-parturient period, identifying critical stages or management factors that may put their well-being at risk. This is crucial for developing informed science-based welfare regulations on breeding dog welfare [17,18,19].

A major component of welfare is affective state (i.e., an animal’s feelings and emotional state [20]), which can be gauged by exposing an animal to a mild stressor and recording their behavioral and physiological response [21]. Previous studies in CB kennels have utilized stranger approach as a mild stressor and found it was successful in eliciting responses ranging from fearful to affiliative [14,15,16,22].

Physiologic responses to stressors can be difficult to explore in this population, as many dogs are not handled extensively outside of basic husbandry and veterinary purposes, and short-term metrics (e.g., plasma, saliva) are susceptible to handling stress [23]. Changes in basal concentrations of dogs’ physiologic metrics throughout the peri-parturient period have previously been evaluated but studies show conflicting findings. For example, multiple studies have measured changes in cortisol throughout various stages of the peri-parturient period, which is a product of hypothalamic-pituitary-adrenal (HPA) axis activation and can be used to capture changes in an individual’s stress response [24,25]. They found no significant changes in plasma, hair, or salivary cortisol [26,27,28]. However, a recent study identified a trend of increase in hair and claw cortisol from mating to 60 days postpartum [29]. Explanations for the differences between studies may include sample size, breed, and method of cortisol collection, with hair and claw cortisol representing average concentrations over a long period, and plasma and saliva representing short-term concentrations that may be sensitive to handling stress [23,30]. Changes in cortisol throughout the peri-parturient period may be due to mobilization of energy to meet requirements for milk production, although the effect of external stressors cannot be excluded.

Even fewer studies have measured physiologic variables related to immune function throughout the peri-parturient period in dogs. A common, non-invasive measure of immune function is secretory immunoglobulin A (sIgA), which is the primary antibody on mucosal surfaces of most mammals [31]. sIgA aids in maintaining a balanced microbiota, while defending against viruses, toxins, and bacteria [31,32]. sIgA is increasingly used as a welfare indicator, as its concentration can be affected by the intensity and duration of a stressor [33,34]. Grellet et al. [35] examined fecal sIgA concentrations in dogs throughout gestation and lactation, and found concentrations were lower during the second month of lactation as compared to the first month of lactation, throughout gestation, and control animal concentrations. The authors hypothesized the lower concentrations were due to the high milk production and the mobilization of IgA in milk during this time [35]. However, as with other studies, these findings were not related to stress and welfare of dams.

The objective of the current study was to identify changes in physical, behavioral, and physiologic metrics that might be associated with dam welfare throughout the peri-parturient period in CB kennels. As changes in these metrics may also occur due to natural biological changes throughout the peri-parturient period, we consulted previous literature characterizing typical changes during this period to interpret results and hypothesize if any deviations observed in the current study may be due to changes in welfare. We further aimed to identify relationships between physiologic metrics to aid in future metric selection. Based on previously published literature, this is the first study to assess behavior changes and welfare in dams throughout the peri-parturient period.

## 2. Materials and Methods

### 2.1. Ethics Statement

Experimental procedures were approved by the Purdue University Institutional Animal Care and Use Committee. CB kennel owners volunteered for the current study and were told they could withdraw at any point without consequences. They were required to sign a consent form before the study began and were given a unique ID number in the experimenters’ records to maintain confidentiality. If at any point during testing dams showed signs of agitation or aggression (as characterized by continuous stereotypic behavior, multiple escape attempts, or continuous bouts of lunging, barking, or growling) that caused concern for the dam’s or experimenter’s safety, testing was stopped.

### 2.2. Subjects and Facilities

Subjects were 74 dams from eight USDA-compliant CB kennels in Midwestern USA (Indiana and Ohio), with an average age of 2.9 years old (range = 1–6 years old), comprised of 19 breeds or crossbreeds (Supplemental Materials Table S1). There were no breed restrictions. Criteria for dog selection included bitches that were suspected to be less than three-weeks pregnant (as reported by the breeder), physically healthy, and over one-year of age. We collected data from a minimum of six dams per kennel to ensure adequate representation of within kennel variation. The total number of kennels included in the study ensured a representation of different environments and management practices and allowed us to reach a sample size larger than other similar research in different populations [28,29,36]. To maximize data collection time and travel, each kennel was required to have a minimum of three bitches that met selection criteria and were expected to whelp in the same week. Prior to parturition (normally 1 week prior), dams were relocated from their regular pen with conspecifics to a whelping pen where they were housed individually. Whelping pens were partitioned into a nursing area where the litter was contained and an adjacent area accessible to the dam only. Dams were returned to their regular pens with conspecifics after weaning (normally 6 weeks after parturition). Sizing of group and whelping pen areas as well as access to exercise areas varied between and within kennels, but all met or exceeded requirements outlined by the USDA [37]. All dams had continuous access to food and water and were accustomed to receiving treats from their caretakers during routine care.

### 2.3. Study Procedure

To determine feasibility of methodology, a pilot study was completed prior to formal data collection where seven dams were assessed at one CB kennel in Indiana. This data was not included in the current study. Data collection occurred throughout June 2019 to January 2021. To minimize the risk of disease transfer, all experimenters wore disposable boot covers (Innovative Haus, Premium Thick Waterproof Disposable Shoe Covers) over their shoes and donned nitrile gloves (KIMTECH^TM^) prior to kennel entry. This study was a pre-post observational study and dams were assessed at four time-points throughout the peri-parturient period: approximately 6 weeks prepartum, 1 week prepartum, 4 weeks postpartum and 8 weeks postpartum. At each time point the dams were behaviorally assessed using a modified version of the Field Instantaneous Dog Observation Tool Plus (FIDO+) [14,16,22], which is a three-step test measuring a dog’s response to stranger approach and includes a visual assessment of physical health (described below). Two female experimenters deemed similar in appearance (White, blonde, mid to late 20 s) conducted the assessments. Experimenter 1 (AR) tested 44 dams from five CB kennels, and Experimenter 2 (JW) tested 30 dams from three CB kennels. Throughout the entire procedure, a research assistant was standing outside of the dam’s sightline, recording in a spreadsheet (Microsoft Excel) on an iPad (Apple Inc., Cupertino, CA, USA) the scores communicated verbally by the testing experimenter. Additionally, hair for hair cortisol concentration (HCC), and feces for fecal glucocorticoid metabolites (FGM), fecal sIgA, and presence of intestinal parasites were collected at each time point. An overview of time points and metrics collected is outlined in Table 1.

### 2.4. Behavioral and Physical Health Measures

At the beginning of each testing day, dams were locked into the interior portion of a pen (if they had free access to an outdoor run) by a familiar caretaker. At 4 weeks postpartum dams were locked into their pens with their litters. Previous research in the same population of dogs indicated dams’ behavioral responses to a stranger when locked into the interior portion of their home pens did not differ from their responses when they had free access to their outdoor runs [38]. After a three-minute habituation period, the experimenter proceeded to conduct the behavioral portion of the FIDO+, which is a stranger approach test that was originally described by Stella et al. [14]. The test was modified to consist of three steps, as outlined by Barnard et al. [22]. In step one, the experimenter (unfamiliar to the dogs) approached the pen door, turned to the side with knees slightly bent and averted gaze, hand extended toward the pen, and scored the immediate behavioral response of the dog. The experimenter then tossed a treat over the pen door and recorded whether the dog ate it or not. In step two, the experimenter opened the door with the same body language and scored the immediate behavioral response of the dog. The experimenter then offered the dog a treat from their hand and recorded whether the dog ate it or not. In the third step, the experimenter, with the same body language, extended their hand to touch the dog (without stretching or bending over the dog) while offering the dog a treat with the other hand. The experimenter scored the immediate behavioral response of the dog, whether they could touch the dog, and if the dog ate the treat. If the dog was in reach but showed signs of fear or avoidance, touch was scored as ‘no’. As originally outlined by Bauer et al. [16], responses to stranger approach at each step were classified as red, yellow, or green (RYG), which were intended to represent varying degrees of pain, fear, distress, and other negative states as well as positive states of being. Red responses were those indicative of fear and may have included stereotypic behaviors (e.g., circling and pacing), aggression (e.g., lip lifting, lunging, hard body language), calming signals (e.g., yawning, lip licking, body shake, paw lifting), attempting to flee, or freezing. Yellow responses were those that involved conflicting behaviors such as approaching and avoiding the stranger, ambivalent body language, or any behavior that did not fall into either green or red categories. Green responses were those that included affiliative or neutral behaviors such as approach or soliciting attention with a relaxed body language, or exhibition of maintenance behaviors (e.g., eating, drinking, resting). Experimenters recorded if dams ate the offered treats (1cm pieces of Canine Carry Outs- Beef Flavor or Pup-Peroni- Original- Beef Flavor, Big Heart Pet Inc.) and whether they exhibited aggressive and/or stereotypic behavior or if they were frantic/overstimulated throughout the test. For reliability analysis, behavioral testing at 6 weeks prepartum was recorded using a portable camcorder (Sony Handycam HDR-CX405, mounted on a tripod). The visual physical health assessment was completed after hair collection. Dams were routinely examined by a veterinarian and inspected by the breeders, and they reported to us if any major health issues were raised. The visual health assessment allowed for the collection of key physical health indicators in a non-invasive, non-stressful way. This assessment has been previously validated in this population of dogs, demonstrating a high agreement with hands-on physical exams [14]. The physical health assessment was identical to that used by Bauer et al. [16] and Stella et al. [14] and was scored at every time point as follows: body condition score (BCS) on a scale of 1–5 (1 = emaciated, 2 = thin, 3 = moderate, 4 = stout, 5 = obese) except at 1 week prepartum when dams were heavily pregnant, body cleanliness as measured by percentage of body covered in debris (0%, 1–25%, 26–50%, 51–75%, >76%), tear staining (none, mild, moderate, severe), and presence or absence of nasal discharge, ocular discharge, sneezing, coughing, missing fur or poor coat, wounds, sores or lesions, and lameness.

### 2.5. Physiologic Measures

#### 2.5.1. HCC

At all time points, hair collection occurred after the behavioral testing was complete. To ensure low-stress handling, caretakers were asked where dams commonly underwent veterinary procedures and/or grooming, and collection occurred in the same location. Common places included in the dam’s pen or a quiet front room of the kennel. Dams were offered baby food puree (Turkey, Ham, or Chicken and Gravy; Gerber, Nestle) on a tongue depressor, disposable plate, or spatula throughout collection.

The familiar caretaker placed dams on the floor or a raised surface (i.e., grooming table or bench) with a non-slip surface (if not available, dogs were placed on a non-slip rubber mat) to collect hair samples. Experimenters used electric clippers (Wahl Professional Arco Pet Cordless Clipper) and attempted to collect 50 mg of hair from dams’ lower lumbar area (i.e., directly above the base of their tail). At 1 week prepartum, 4 weeks postpartum and 8 weeks postpartum, hair was only collected from the area shaved at the previous time point (i.e., shave-re-shave technique). Experimenters frequently shaved a larger area than what was needed for 50 mg after samples had been collected to ensure enough regrowth at the subsequent time point. The collected hair was stored in a small, labelled manila envelope, sealed with tape. Dams were placed back in their home pens after collection. To prevent contamination, clippers were cleaned and disinfected between dogs. Hair samples were stored in a cool, dry area until ready to be shipped to the laboratory for analysis in two batches.

Cortisol levels in hair samples were determined by enzyme immunoassay (EIA) (Salimetrics, Carlsbad, CA, USA) in the Endocrine Technologies Core (ETSC) at the Oregon National Primate Research Center (ONPRC) using a modification of an existing protocol [39]. Hair samples were washed with isopropanol, drained through P8 filter paper (Thermo Fisher Scientific, Waltham, MA, USA) and dried. Approximately 50 mg of each washed and dried hair sample was weighed into a reinforced microtube, and five 4 mm steel beads were added for grinding. Samples were then ground (2 × 5 min) using a Spex SamplePrep 5100 grinder (Spex, Metuchen, NJ, USA). One ml of methanol was added to ground hair samples and cortisol was extracted overnight with gentle shaking. Samples were then centrifuged to collect hair and supernatants were transferred to fresh tubes and evaporated to dryness. Samples were reconstituted in 0.3 mL of PBS and cortisol levels determined by EIA. Recovery was determined at the same time as sample analysis and used to adjust final sample cortisol values. Inter-assay variation was 8.5%. Intra-assay variation for the first batch was 3.0%, and the second batch was 8.4%.

#### 2.5.2. FGM, Fecal sIgA, and Presence of Parasites

Caretakers were instructed to collect spontaneously voided feces from dams the night before or morning of each testing day and store them in a cool place in labeled Ziploc bags. Variation in collection time would not have affected results, as the metric was intended to represent a basal concentration as opposed to a baseline before or in response to a specific stressor. Moreover, as FGMs represent average cortisol concentrations over approximately 24 h in dogs, diurnal fluctuations in concentrations were not a concern [23,40]. When dams were group-housed, breeders typically isolated the dam in a pen or outside yard to obtain feces. Isolation for a short period of time would likely not affect results, as FGMs are resistant to handling stress and short-term fluctuations in cortisol [23]. Once collected, the experimenters split the fecal sample into two parts, one of which was utilized for FGM and fecal sIgA analyses, and the other for parasite analyses. Limitations of this method are mentioned in the discussion section below. While travelling to and from breeders, feces were stored in Styrofoam coolers with ice packs and/or a refrigerator (approximately 1–3 °C). This ranged from approximately 4 to 48 h. Once transported, feces for FGM and fecal sIgA analyses were stored in a freezer (−20 °C) until shipping to the laboratory, and feces for intestinal parasite analyses were stored in a refrigerator (4 °C) and analyzed on site within one week.

#### 2.5.3. FGM and Fecal sIgA Sample Processing

Fecal samples were shipped on dry ice to Omaha’s Henry Doorly Zoo and Aquarium Endocrinology Lab in two batches. Samples were thawed and processed separately for cortisol and sIgA. Processing for cortisol involved drying > 1 g of feces in a 60 °C oven for 48–72 h to account for variation in fecal water content. Dried samples were pulverized into a fine power and 0.5 g was extracted by mechanically shaking overnight in 5 mL of 80% *v*:*v* methanol. Samples were centrifuged at 3000× *g* for 15 min and the supernatant stored at −20 °C until analysis. Processing for sIgA involved weighing 0.5 g of wet feces, adding 1.5 mL of PBS (524650, EMD Millipore, Burlington, MA, USA) containing protease inhibitor (cOmplete mini [1 tablet/10mL PBS], Roche, Basel, Switzerland), vortexing for 30 s and incubating 60 min at RT prior to a double centrifugation step (3000× *g* 15 min). The supernatant was collected and frozen until sIgA analysis within 1 month.

#### 2.5.4. Cortisol and sIgA Immunoassays

##### Cortisol Enzyme Immunoassay

Fecal cortisol concentrations were quantified by EIA using an anti-cortisol antiserum (R4866) and cortisol-horseradish perioxidase (HRP) ligand (C. Munro, University of California, Davis, CA, USA). The polyclonal antiserum raised in rabbits was directed against cortisol-3-carboxymethyloxime (CMO), linked to bovine serum albumin and was shown to cross react with cortisol (100%), prednisolone (9.9%), prednisone (6.3%), cortisone (5%) and <1% with adrostenedione, androsterone, corticosterone, desoxycorticosterone, 11-desoxycortisol, 21-desoxycortisone and testosterone [41]. The EIA was performed according to the methods established by Munro and Lasley [41]. Absorbance was measured at 405 nm (SpectraMax ABS, Molecular Devices, San Jose, CA, USA) and data extrapolated via 4-parameter curve fit using Softmax Pro 7.1 (Molecular Devices). Results of each sample were expressed as ng of cortisol per gram of dry mass feces (dmf).

The cortisol EIA was validated for canine fecal samples by demonstrating parallelism between the standard curve and serial dilutions of pooled fecal extracts (1:4–1:1024) using the first batch. Fecal extracts were run at a 1:50 dilution in EIA buffer. If sample results indicated binding was not within the linear portion of the standard curve, samples were diluted accordingly (1:20 to 1:60). Parallelism results for cortisol were plotted as percent binding versus standard mass. Differences between slopes of the binding curves for the serially diluted canine fecal extract pool and the standard curve was assessed with an F-test. There was no significant difference in slopes between cortisol standards and serially diluted canine fecal samples (F_1,16_ = 0.0417, *p* = 0.841). Inter-assay coefficients of variation for the cortisol assay were 3.9% and 5.4% for internal controls at 54% (20 pg/well) and 33% (70 pg/well) percent binding, respectively, for the first batch, and 5.5% and 7.7% for internal controls at 53% (25 pg/well) and 32% (75 pg/well), respectively, for the second batch.

##### sIgA Enzyme-Linked Immunosorbant Assay

Fecal sIgA concentrations were quantified by a commercial enzyme-linked immunosorbant assay (ELISA) (E40-104-26, E101, Bethyl Laboratories, Montgomery, TX, USA) as follows: 96-well plates were coated for 1 h at RT with a 1:100 dilution of affinity purified goat anti-canine IgA antibody in 100 µL of coating buffer (0.05 M Carbonate-Bicarbonate, pH 9.6). Following coating, plates were washed five times (50 mM Tris, 0.14 M NaCL, 0.05% Tween 20, pH 8.0) before adding 200 µL/well of blocking solution (50 mM Tris, 0.14M NaCL, 1% BSA, pH 8.0) and storing overnight at 4 °C. The next day, plates were washed five times before adding 100 µL/well of duplicate standards (15.6–1000 ng/mL) or samples and incubating 1 h at RT on a light-protected plate shaker (600 rpm). Plates were then washed an additional five times prior to adding 100 µL/well of a 1:75,000 dilution of goat anti-canine IgA:HRP in ELISA buffer (50 mM Tris, 0.14 M NaCL, 1% BSA, 0.05% Tween 20) and incubating for 1 h at RT on a light-protected plate shaker (600 rpm). A final wash step was performed before adding 100 µL/well of 3,3′,5,5′-tetramethylbenzidine (TMB) substrate solution and incubating the plate for 15 min at RT on a light-protected plate shaker (600 rpm). The reaction was stopped with 100 µL/well of 0.18 M H2S04 and absorbance was measured at 450 nm (SpectraMax ABS, Molecular Devices) with data extrapolated via 4-parameter curve fit using Soft-Max Pro 7.1 (Molecular Devices). Results for each sample were expressed as mg of sIgA per gram (wet weight) of feces.

The IgA ELISA was validated for canine fecal samples by demonstrating parallelism between the standard curve and serial dilutions of pooled fecal extracts (1:1000–1:40,000). Fecal extracts were run at a 1:10,000 dilution in ELISA buffer. If samples results were outside of the standard range, samples were diluted accordingly (1:5000 to 1:20,000). Parallelism results for sIgA were plotted as optical density versus standard mass. Differences between slopes of the binding curve for the serially diluted canine extract fecal pool and the standard curve was assessed with an F-test following log transformation of the data. There was no significant difference in slopes between canine sIgA standards and serially diluted canine fecal samples (F_1,13_ = 0.350, *p* = 0.564).

#### 2.5.5. Presence of Intestinal Parasites

The canine fecal samples were stored at 4 °C until a centrifugal fecal floatation assay using Sheather’s sugar solution (SPG 1.25) could be performed to detect the presence of parasite ova and/or cysts. One gram of the feces was placed in a paper cup containing 15–20 mL of the flotation solution and mixed thoroughly. This mixture was strained through a cheesecloth, and the solution was collected into a 15 mL centrifuge tube until it formed a convex meniscus. A cover glass was placed on this fluid meniscus and gently pressed-down such that it was securely seated on the rim of centrifuge tube. This centrifuge tube with cover glass on it was spun at 1500 rpm for 7 min in a swinging head centrifuge. After the centrifugation was complete, the cover glass was gently removed straight up, placed on a microscopic slide, and examined under 10× objective lens of a compound microscope to detect parasite eggs/oocysts/cysts [42]. If a sample was positive for *Cryptosporidium*, the diagnosis was confirmed using *Cryptosporidium*/*Giardia* fecal direct fluorescent antigen detection kit (IVD Research Inc., Carlsbad, CA, USA).

### 2.6. Deviation of Testing Date from Ideal Time Point and Data Point Exclusion

Due to unanticipated whelping dates and time constraints, the days in which dams were tested may have deviated from ideal time points. Average deviations were as follows (ideal time point ± mean deviation): 6 weeks prepartum ± 5 days (range = 7-weeks to 4-weeks and 2 days prepartum), 1 week prepartum ± 3 days (range = 2 weeks prepartum to 1 day before parturition), 4 weeks postpartum ± 3 days (range = 3-weeks to 5-weeks and 2 days postpartum), and 8 weeks postpartum ± 2 days (range = 7-weeks and 5 days to 9-weeks and 1 day postpartum). To ensure the ranges of time in which each ideal time point was tested did not encompass a significant behavioral or physiological change that would skew results, we consulted previous literature on this topic.

Studies indicate that naturally occurring hormones (i.e., progesterone, estrogen, prolactin, etc., which may impact behavior and physiologic metrics measured) and immune function throughout the peri-parturient period remain relatively stable during the ranges we tested at 6 weeks prepartum and 4 and 8 weeks postpartum [43,44,45]. Furthermore, literature suggests no significant change in cortisol throughout the ranges tested at 6 weeks prepartum and 4 weeks postpartum [26,27]. Changes in cortisol concentrations throughout the range tested for 8 weeks postpartum have not been identified in previous literature, so there is no basis for exclusion of data points during that time. Throughout the range tested at 1 week prepartum, daily changes in naturally occurring hormones (i.e., progesterone, estrogen) occur [43]. However, even the most extreme values (i.e., those tested the furthest away from and closest to parturition) during this time are dramatically different than they would be during any of the other time points tested [43], allowing for detection of differences between time points.

**Figure 1:**
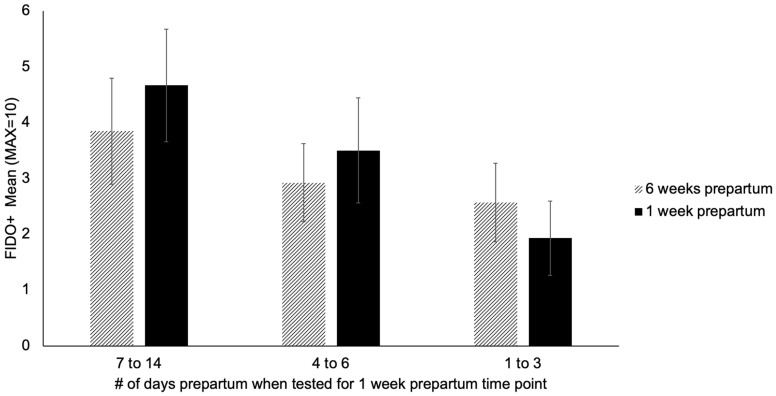
Mean FIDO+ score at 6-weeks and 1 week prepartum separated by number of days prepartum when tested at the 1 week prepartum time point.

In regards to the dams tested in the three days before parturition, literature indicates a peak in cortisol concentration 8–24 h before whelping [26,46], however our physiologic metrics would not have captured this peak since both hair and fecal samples are representative of longer-term changes. Regarding behavior during this period, literature suggests that 1–3 days prior to parturition bitches may exhibit restlessness, seek seclusion, or seek attention from a caregiver [47,48]. To ensure that dams tested within this window would not skew data, we visually examined their FIDO+ scores (*n* = 13) compared to those dams tested 4–6 days prepartum (*n* = 14) and 7–14 days prepartum (*n* = 16). We found that those tested 7–14 days before parturition had the highest (i.e., most affiliative) FIDO+ scores, followed by those tested 4–6 days before, and then 1–3 days before (Figure 1). When the FIDO+ scores for the same groups were examined at the 6 weeks prepartum time point, they followed the same trend (Figure 1). Further, the 6 weeks prepartum scores were similar to the 1 week prepartum scores, with the difference not being biologically meaningful (Figure 1). Ultimately, the lower FIDO+ scores for the group of dams tested 1–3 days prepartum appeared to be associated with the general fear level of those individual dams and not with the timing of testing, hence not justifying exclusion from the sample. Therefore, based on evidence from previous literature and visual analyses of data, no data points were removed from analyses due to deviation of testing date from ideal time point.

### 2.7. Statistical Analyses

Statistical analyses were performed using R version 4.1.1 (R Core Team, Vienna, Austria) alongside RStudio version 2021.9.0.351 (RStudio Team, Boston, MA, USA) with α ≤ 0.05.

#### 2.7.1. Inter-Rater Reliability (IRR)

IRR was calculated between Experimenter 1 and 2 for the behavioral portion of the FIDO+. Each experimenter scored the same set of 30 dogs from videos, nine of which were also included in the final analyses of the current study. As outcome variables were ordinal (i.e., RYG), IRR was calculated for the approach, open, and reach steps of the FIDO+ using intraclass correlations (ICC) implemented by the package *DescTools* [49]. Koo & Li’s [50] interpretation of ICC values was utilized: ≤0.5 = Poor, 0.5–≤0.75 = Moderate, 0.75–≤0.90 = Good, ≥ 0.90 = Excellent. As the touch step of the FIDO+ had a nominal, binary outcome (i.e., yes or no), IRR was calculated using Cohen’s Kappa in the *irr* package [51]. Kappa results were interpreted using the guidelines provided by McHugh [52]: 0–0.20 = None, 0.21–0.39 = Minimal, 0.40–0.59 = Weak, 0.60–0.79 = Moderate, 0.80–0.90 = Strong, > 0.90 = Almost Perfect.

#### 2.7.2. Changes in Behavior, HCC, FGM, and Fecal sIgA

Scores from the FIDO+ (outlined above in Section 2.4) were converted to points as follows: Red = 0, Yellow = 1, Green = 2, Treat = 1, No treat = 0, Touch = 1, No touch = 0. Points were summed (maximum of 10) and the value was used as a final behavior score for each dam at each time point. Separate analyses for treat consumption (maximum of 3 points) and behavior (i.e., approach, open, reach and touch steps- maximum of 7 points) throughout the FIDO+ were also conducted. The formula, limit of detectability (LOD)/2, was utilized to obtain a result for hair samples with HCCs below the LOD (which was variable between samples as weight was a factor; *n* = 5), as the data were skewed [53,54]. Significant changes in behavior, HCC, FGM and fecal sIgA were identified using linear mixed-effects models implemented by the *nlme* package [55] and Wald tests by the *car* package [56]. Age (three categories: 1–2, 3–4, and 5–6 years old) and time point (6 weeks prepartum, 1 week prepartum, 4 weeks postpartum and 8 weeks postpartum) were entered in the model as fixed effects, and dog ID nested within kennel ID was included as a random effect to account for non-independence across time points and kennel effects. For all models, residuals were checked for normality using histograms and QQ plots, and heteroscedasticity was examined by plotting residuals against fitted values. Right-skewed data were log-transformed where appropriate. Restricted maximum likelihood (REML) was employed in all models. If significant, the *lsmeans* function with a tukey *p*-value adjustment for multiple comparisons, implemented by the *lsmeans* package [57], was used to run pairwise comparisons.

#### 2.7.3. Relationship between Physiologic Metrics

Normality of variables were checked using histograms, QQ plots, and Shapiro-Wilk tests. All variables were log-transformed to correct right-skewedness. As variables were not independent of each other due to repeated measures, a repeated measures correlation from the package *rmcorr* [58] was utilized to determine the correlation between pairs of physiologic metrics.

#### 2.7.4. Parasites and Physical Health

Descriptive statistics (i.e., means and proportions) were derived to depict the distribution of physical health scores and presence of intestinal parasites.

## 3. Results

Fifteen dams were excluded from the final analyses due to various reasons such as lack of pregnancy, birthing no living puppies, or issues with travel caused by actual whelping dates differing from those predicted. The final analyses included 59 dams with a mean age of 2.9 years old (range = 1–6 years old), comprised of 18 breeds or crossbreeds (Supplemental Materials Table S1). Sample sizes for each metric at each time point are listed in Table 2. Missing data occurred due to various reasons such as technological errors, timing issues, lack of spontaneous defecation, lack of hair regrowth, and disruptions due to the COVID-19 pandemic. Weaning dates were not provided for 29 out of the 58 dams tested at the 8-week time point, however, the 29 dates provided revealed a mean weaning date of 6-weeks and 6 days postpartum (range = 6-weeks to 8-weeks and 1 day postpartum). One dam was weaned the day of testing. This dam and the dams with no weaning date provided were not excluded from the analyses as it was likely weaning had begun to occur naturally [59,60].

### 3.1. IRR

As a fixed set of multiple raters (k = 2) scored each video, ICC3k was reported [61]. The IRR between Experimenter 1 and 2 for approach was moderate (ICC3k = 0.65, *p* = 0.003, 95% CI [0.645, 0.661]), open was good (ICC3k= 0.83, *p* < 0.001, 95% CI [0.827, 0.835]), and reach was good (ICC3k = 0.85, *p* < 0.001, 95% CI [0.844, 0.851]). The IRR for touch was moderate (Kappa = 0.74, *p* < 0.001).

### 3.2. Changes in Behavior, Treat Consumption, HCC, FGM, and Fecal sIgA

**Figure 2:**
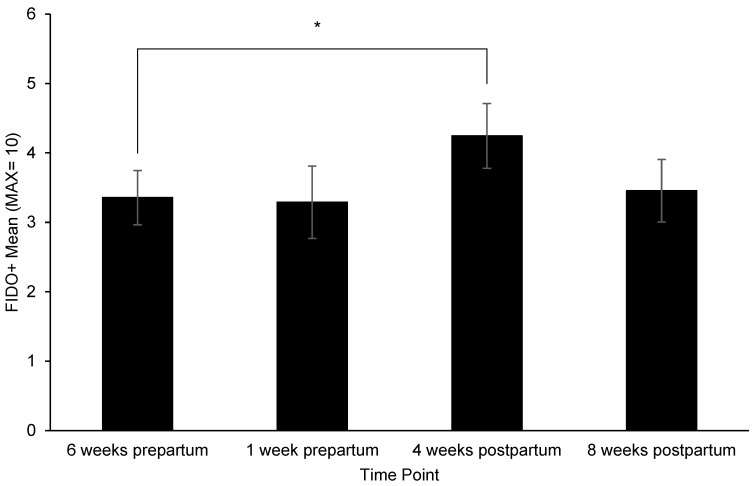
Change in Field Instantaneous Dog Observation Tool + (FIDO+) response across time points. Sample sizes for each time point were *n* = 59, *n* = 38, *n* = 57, and *n* = 55, respectively. Error bars represent SE. * indicates *p* ≤ 0.05.

**Figure 3:**
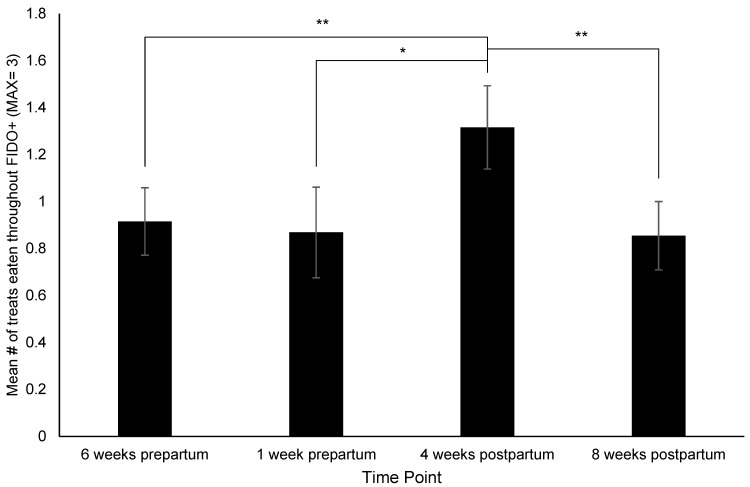
Change in treat consumption throughout the Field Instantaneous Dog Observation Tool + (FIDO+) across time points. Sample sizes for each time point were *n* = 59, *n* = 38, *n* = 57, and *n* = 55, respectively. Error bars represent SE. * indicates *p* ≤ 0.05, ** indicates *p* < 0.01.

**Figure 4:**
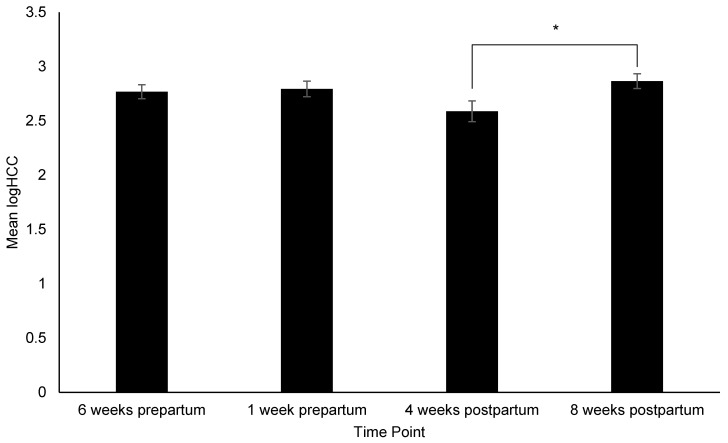
Change in hair cortisol concentration (logHCC) across time points. Sample sizes for each time point were *n* = 59, *n* = 43, *n* = 41, and *n* = 53, respectively. Error bars represent SE. * indicates *p* ≤ 0.05.

**Figure 5:**
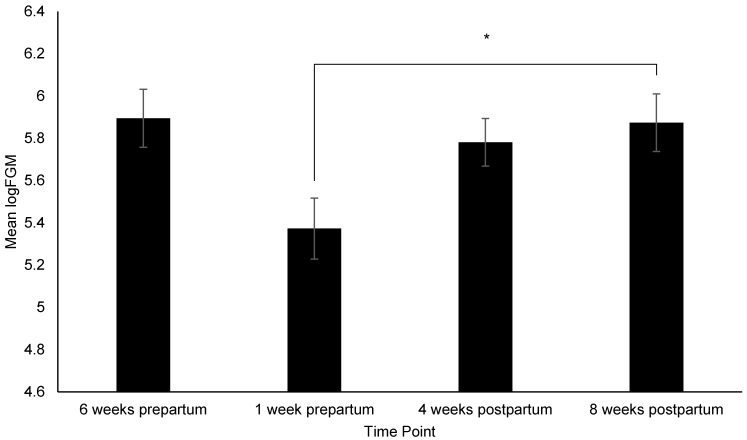
Change in fecal glucocorticoid metabolites (logFGM) across time points. Sample sizes for each time point were *n* = 34, *n* = 37, *n* = 53, and *n* = 52, respectively. Error bars represent SE. * indicates *p* ≤ 0.05.

**Figure 6:**
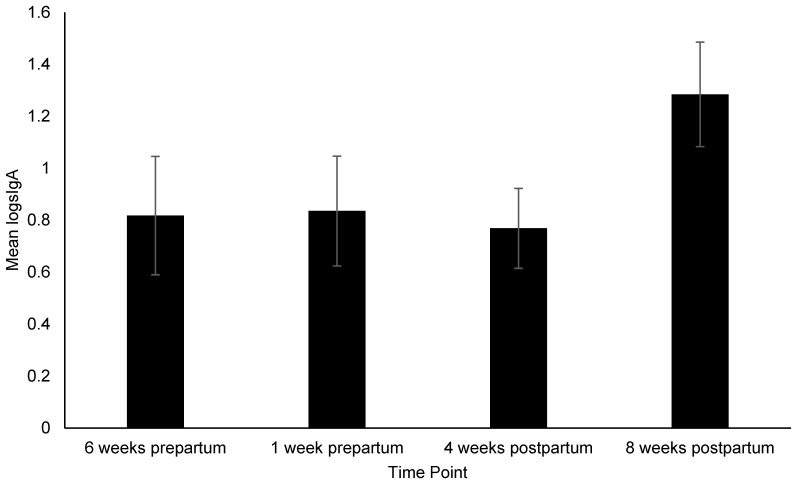
Change in fecal secretory immunoglobulin A (logsIgA) across time points. Sample sizes for each time point were *n* = 34, *n* = 37, *n* = 53, and *n* = 51, respectively. Error bars represent SE.

The main effect of age or its interaction with time point was not significant in any model; therefore, it was removed to improve model fit. There was a significant effect of time point on FIDO+ score (Χ^2^_(3)_ = 8.41, *p* = 0.04), treat consumption during the FIDO+ (Χ^2^_(3)_ = 15.95, *p* = 0.001), HCC (Χ^2^_(3)_ = 9.14, *p* = 0.03) and FGM (Χ^2^_(3)_ = 8.49, *p* = 0.04) (Supplemental Materials Table S2). Pairwise comparisons identified the following: Dams had significantly higher (i.e., more affiliative) FIDO+ scores at 4 weeks postpartum than 6 weeks prepartum (*p* = 0.03) (Figure 2), consumed more treats during the FIDO+ at 4 weeks postpartum than 6-weeks (*p* = 0.008) and 1 week prepartum (*p* = 0.05), and 8 weeks postpartum (*p* = 0.005) (Figure 3), exhibited a significant increase in HCC from 4 to 8 weeks prepartum (*p* = 0.02) (Figure 4), and a significant increase in FGM from 1 week prepartum to 8 weeks postpartum (*p* = 0.04) (Figure 5) (Supplemental Materials Table S3). No significant effect of time point on fecal sIgA (Χ^2^_(3)_ = 6.56, *p* = 0.09) (Figure 6) or the behavioral portion of the FIDO+ (i.e., approach, open, reach and touch steps) (Χ^2^_(3)_ = 4.02, *p* = 0.26) were identified (Supplemental Materials Table S2).

### 3.3. Relationship between Physiologic Metrics

There was a significant positive correlation between FGM and fecal sIgA (r_rm_ = 0.23, *p* = 0.002). No significant correlation was identified between HCC and FGM (r_rm_ = −0.14, *p* = 0.17), or HCC and fecal sIgA (r_rm_ = −0.05, *p* = 0.65).

### 3.4. Parasites and Physical Health

At each respective time point the percentage of dams with intestinal parasites was 11%, 4%, 23%, and 15% (Table 3). In order of prevalence, intestinal parasites detected were *Giardia* (*n* = 12), *Cystoisospora* (*n* = 9), *Trichuris* (*n* = 1), and *Cryptosporidium* (*n* = 1). If a *Giardia* infection was detected, kennel hygiene and sanitation measures were recommended to breeders to avoid the spread of infection. Of those dams who were tested for intestinal parasites at both 4 and 8 weeks postpartum (*n* = 41), only 5% (*n* = 2) were positive at both time points.

Changes in physical health observed over the peri-parturient period are in Table 4. Throughout the four time points, 38 dams had a cleanliness score of 1–25%, 7 dams had a score of 26–50%, and the remainder had a score of 0%. No dam had a cleanliness score over 50% (i.e., 50% of body or over covered in debris). Poor coat condition was observed twice due to missing hair and dandruff. Lameness was observed once. Nasal discharge, sneezing, coughing, or wounds were not observed.

## 4. Discussion

Based on previously published literature, this is the first time a study has examined changes in behavior and other metrics that may be indicative of welfare throughout the peri-parturient period in dogs. Most significant changes in physiological and behavioral metrics were likely due to natural biological changes that occur during this time. However, some changes deviated from what one would expect based on previous literature, therefore requiring further investigation as to their effect on dam welfare as discussed below.

### 4.1. Behavior

Dams exhibited more affiliative responses at 4 weeks postpartum compared to 6 weeks prepartum, which may have occurred for several reasons. Dams consume 2.5 to 3 times their normal energy requirements during the third and fourth week of lactation [62], and because our behavioral test involved giving treats to them, it is likely that food rather than a change in affective state may have enticed the dam to interact with the experimenter. This is further supported by the fact that dams consumed significantly more treats at 4 weeks postpartum than all other time points, and there was no significant change in the behavioral portion of the FIDO+ across time points. The effect of food motivation on performance in behavioral tests has previously been observed in the same population [22]. Furthermore, dams have high concentrations of oxytocin and prolactin during lactation, which have anxiolytic effects [63]. These hormones may have attenuated dams’ fear responses to the mild stressor of an unfamiliar person, leading to an increase in affiliative behavior. Finally, dams may have exhibited increased affiliative behavior due to environmental and/ or management changes. At 6 weeks prepartum most dams were housed in pens with conspecifics, where they frequently experienced high levels of noise due to barking, and less one-on-one interaction with caretakers. As demonstrated in animal shelters, high levels of noise can have detrimental consequences for dogs’ physical and behavioral health [64,65]. In contrast, at 4 weeks postpartum, dams were housed individually in whelping pens with their litters, typically in a separate room or wing of the building from their home pen as to provide a quiet environment, and experienced an increase in one-on-one interactions with caretakers. If positive, these repeated one-on-one interactions may have increased approach and affiliation not only towards the caretaker but unfamiliar people as well [66,67,68]. Thus, the decreased noise and increased one-on-one interactions experienced at 4 weeks postpartum may have positively influenced dams’ behavioral responses to an unfamiliar person.

### 4.2. HCC

The most parsimonious explanation for the increase in HCC from 4 to 8 weeks postpartum, which, due to its representation of concentrations over weeks to months [23], encompasses an increase occurring between the month prior and the month following the 4 weeks postpartum time point, may involve some of the same underlying mechanisms. As previously mentioned, dams have elevated energy requirements during lactation, and the mobilization of energy is associated with increased basal concentrations of glucocorticoids [69,70]. Although a different species, our finding is supported by a recent study in cats, which observed higher concentrations of serum cortisol at 4 weeks postpartum than before mating, 1–2 days after parturition and 8 weeks postpartum [36]. In contrast, more recent studies in dogs found no change in coat and claw cortisol between 4 and 8 weeks postpartum [29], or plasma and salivary cortisol between parturition, 21, and 60 days postpartum [28]. The difference in findings may be due to the larger sample size and representation of multiple breeds in the current study. Furthermore, although not statistically significant, the observed decrease in HCC from 1 week prepartum to 4 weeks postpartum may warrant further investigation. One would expect that due to increased energy mobilization and the spike in cortisol concentration that occurs 8–24 h before whelping [26,46], as well as potential hypo-responsiveness of the basal HPA axis at 1 week prepartum [69], concentrations would be higher at 4 weeks postpartum. The fact that we did not observe this may imply that environmental and/or management factors, such as movement to the whelping pens, may be influencing the long-term activation of dams’ stress responses.

### 4.3. FGM

The observed increase in FGM from 1 week prepartum to 4 weeks postpartum is also likely due to natural biological changes. We discussed how basal concentrations of glucocorticoids are increased during lactation, and that increase is likely intensified due to low activation of the basal HPA axis at 1 week prepartum [69]. However, because FGM concentrations did not decrease after puppies were weaned at 8 weeks postpartum, non-physiologic mechanisms may be involved. For example, the 8 week postpartum period coincides with the potential psychological stressor of weaning, and the change of environment and management when dams are moved back to their regular pens with conspecifics. It is also likely that dams engaged in more physical activity at this time point than at 1 week prepartum and 4 weeks postpartum, as they had more area to move, and they had indoor/ outdoor access and conspecifics to interact with in their regular pens as compared to whelping pens. Therefore, this and the finding that FGM concentrations at 8 weeks postpartum were not significantly different than those at 6 weeks prepartum suggest that arousal may have accounted for the increase in glucocorticoids [71,72].

### 4.4. sIgA and Intestinal Parasites

No significant effect of time on fecal sIgA concentration was identified. However, the pattern of mean values across time points is consistent with previous literature. Progesterone, which occurs in high concentrations during gestation [43], is associated with immunosuppression, which is consistent with the low levels of fecal sIgA observed throughout gestation [44,73]. The concentrations remained low at the 4 week postpartum time point, likely due to transfer of immunity from the dam to puppies via milk [35,44]. Although not significant, the sharp increase in fecal sIgA concentration at 8 weeks postpartum is consistent with previous literature citing immune responsiveness increasing after termination of lactation [45]. This may be supported by the percentage of dams with parasites decreasing from 23% to 15% from 4 to 8 weeks postpartum and only 5% of dams testing positive at both time points. However, we cannot rule out that antiparasitic agents were administered between the two time points as this information was not collected. As this topic is widely understudied in CB kennels, future studies should identify additional variables (e.g., diet, medical intervention, sanitation, vitamins and supplements, deworming protocols, etc.) that may affect presence of intestinal parasites in CB kennels and determine how they affect dam and puppy welfare. The increase at 8-weeks may also indicate acute stress [33,74], which is supported by no significant decrease in FGM from 4 to 8 weeks postpartum, and may be due to stress of weaning, or environmental and management changes. This time point is also associated with the lowest percentage of 0% cleanliness scores (67.3%), further supporting the idea that dams were experiencing some distress and potentially decreased welfare.

### 4.5. Limitations

The current study’s design (i.e., a pre-post observational study) did not allow us to determine the causality of the changes observed. Further, it is important to note that the heterogeneity of fecal samples due to collection methods (i.e., using half of the sample for parasites and the other half for fecal sIgA and FGM analyses before homogenizing the sample) may have affected fecal sIgA and FGM concentrations. Literature in other species determined that sIgA and cortisol are not evenly distributed throughout fecal masses and therefore should be collected in their entirety and homogenized before analyses [75,76,77]. Future studies should examine patterns of sIgA and cortisol distribution throughout fecal masses in dogs to inform collection methods, as heterogeneity does not occur in every species [77]. Another limitation of the current study is the possibility that familiarity of the FIDO+ and/or experimenter over time affected dams’ behavioral responses. However, this is unlikely, as recently published literature in the same population found behavioral scores across three consecutive days of testing were highly correlated [22], and dams’ scores decreased (i.e., became less affiliative) from 4 to 8 weeks postpartum. Additional limitations of the current study include missing data points and deviation of testing dates from ideal time points. Finally, volunteer bias may have been present in the current study, which may compromise the ability to generalize our results to the entire CB population. Although not a limitation, it is worth mentioning that as a high degree of variability likely exists between CB and other populations, the results cannot be said to represent all dams throughout the peri-parturient period.

### 4.6. Implications and Future Directions

The significant positive correlation between fecal sIgA and FGM may have implications for strategic welfare assessment metric selection. Animal studies reveal conflicting results regarding the interplay between glucocorticoids and sIgA, as they have identified a negative relationship [78,79], a positive relationship [80], and no relationship [81,82,83] between the two variables. This is likely because IgA concentrations vary in a non-consistent manner, according to the intensity and duration of stressor involved [33]. Theoretically, sIgA increases in response to acute stressors, making it plausible that FGM and sIgA were both capturing responses to acute stress in the current study, whereas HCC was capturing chronic stress [23,33]. However, our finding should be interpreted with caution, as changes related to the peri-parturient period (i.e., immune reactivity, glucose metabolism, etc.) may have affected concentrations and we do not have any information about stressors that may have occurred during collection windows. Further, as mentioned above, our methodology for fecal collection had limitations. Regardless, our data adds to the literature on this topic and may have implications for reducing the number of metrics collected in dogs to ensure welfare while accommodating budget and time constraints.

The current study provides the basis for future research on environmental and management factors that may affect dam welfare throughout the peri-parturient period in CB kennels, such as timing and management changes related to moving dams to whelping areas. Improving dam welfare whilst in CB kennels may have implications for puppy welfare, such as improved ability to cope with typical stressors such as transportation, transitioning to a new home, and potentially becoming breeding bitches themselves. Further, in-kennel welfare improvement may subsequently help dams cope with future stressors, such as encountering novel social and non-social stimuli if rehomed after their breeding careers.

## 5. Conclusions

It appears that most changes in metrics that were recorded throughout the peri-parturient period are due to naturally occurring biological changes. For example, increases in HCC and FGM throughout lactation are likely due to the mobilization of resources to fulfill energy requirements, and the increase in affiliative responses at 4 weeks postpartum could be a consequence of increased energy requirements and hormonal changes. However, some changes such as the decrease in HCC from 1 week prepartum to 4 weeks postpartum and the lack of change between FGM between 4 and 8 weeks postpartum were not in line with natural biological changes that occur during this time. Therefore, it is plausible that typical management practices and environmental changes such as moving to whelping pens and weaning or moving back to group housing may have facilitated the variations in physiologic metrics observed. Further, as dams became more affiliative at 4 weeks postpartum, more positive one-on-one interactions from their caretaker may have influenced their responses to an unfamiliar person. Given the scarcity of data on this topic, whether these deviations are impacting dam welfare and to what extent various management practices can modulate concentrations needs further investigation. Therefore, future research should investigate changes in environmental and management factors to understand how they influence dam welfare throughout the peri-parturient period in CB kennels. This research has important implications for dam and puppy welfare when coping with future stressors and informing welfare regulations for breeding bitches.

## Figures and Tables

**Table 1:** Data collection time points and metrics collected.

Time Point	Metrics Collected
6 weeks prepartum	FIDO+
	HCC
	FGM
	Fecal sIgA
	Presence of intestinal parasites
	Physical health assessment
1 week prepartum	FIDO+
	HCC
	FGM
	Fecal sIgA
	Presence of intestinal parasites
	Physical health assessment
4 weeks postpartum	FIDO+ (with litter present)
	HCC
	FGM
	Fecal sIgA
	Presence of intestinal parasites
	Physical health assessment
8 weeks postpartum	FIDO+
	HCC
	FGM
	Fecal sIgA
	Presence of intestinal parasites
	Physical health assessment

**Table 2:** Sample Sizes for each variable and time point.

Metric	6 Weeks Prepartum	1 Week Prepartum	4 Weeks Postpartum	8 Weeks Postpartum
FIDO+	*n* = 59	*n* = 38	*n* = 57	*n* = 55
HCC	*n* = 59	*n* = 43	*n* = 41	*n* = 53
FGM	*n* = 34	*n* = 37	*n* = 53	*n* = 52
Fecal sIgA	*n* = 34	*n* = 37	*n* = 53	*n* = 51
Parasites	*n* = 18	n = 27	*n* = 48	*n* = 46

**Table 3:** Classification and number of parasites detected at each time point.

Parasite	6 Weeks Prepartum	1 Week Prepartum	4 Weeks Postpartum	8 Weeks Postpartum
*Cryptosporidium*	0/18 (0%)	0/27 (0%)	1/48 (2%)	0/46 (0%)
*Cystoisospora* ^a^	0/18 (0%)	0/27 (0%)	5/48 (10%)	4/46 (9%)
*Giardia*	1/18 (6%)	1/27 (4%)	7/48 (15%)	3/46 (7%)
*Trichuris* (whipworm)	1/18 (6%)	0/27 (0%)	0/48 (0%)	0/46 (0%)
Total	2/18 (11%)	1/27 (4%)	13/48 (27%) ^b^	7/46 (15%)

^a^ *Cystoisospora canis* (*n* = 2), *Cystoisospora ohioensis* complex (*n* = 6) and species not determined (*n* = 1); ^b^ Two dams were positive for two types of parasites, resulting in 23% of dams with intestinal parasites.

**Table 4:** Dam physical health at each time point.

Metric	6 Weeks Prepartum	1 Week Prepartum	4 Weeks Postpartum	8 Weeks Postpartum
BCS ^a^	3.21	N/A	3.07	3.07
Cleanliness–0% ^b^	86%	85%	77%	67%
Ocular discharge–‘present’ ^b^	7%	5%	11%	9%
Tear staining–‘moderate’ or ‘severe’ ^b^	29%	21%	26%	35%

^a^ Mean; ^b^ Percentage of dams.

## Data Availability

Data is available upon request to the corresponding author.

## Supplementary Materials

The following supporting information can be downloaded at: https://www.mdpi.com/article/10.3390/ani12202820/s1, Table S1: Subjects; Table S2: Linear mixed-effects model output for fixed effects. Statistically significant *p*-values are bolded; Table S3: Pairwise comparison output. Statistically significant *p*-values are bolded.
