# Supplementary material for: Dam (Canis familiaris) Welfare throughout the Peri-Parturient Period in Commercial Breeding Kennels
Source: Animals (Basel). 2022 Oct 18;12(20):2820. doi: 10.3390/ani12202820 (PMC9597717; doi:10.3390/ani12202820)
Supplement: Supplementary file 1 [file animals-12-02820-s001.zip › animals-1854475-supplementary.pdf]

Table S1. Subjects.

Kennel	Dam ID	Dam Breed	Age (years)	Included in final analyses?*
1	001	Dachshund	3	Yes
	002	Dachshund	2	Yes
	003	Maltese/ Yorkshire Terrier	2	Yes
	004	Maltese	2	Yes
	005	Maltese	3	Yes
	006	Maltese	3	Yes
	007	Miniature Poodle	2	Yes
	008	Dachshund	1	No
	009	Dachshund	3	No
	010	Maltese/ Miniature Poodle	1	No
	011	Maltese	2	No
	012	Maltese	1	No
2	013	American Eskimo	2	Yes
	014	American Eskimo	4	Yes
	015	Jack Russell Terrier	1	Yes
	016	Jack Russell Terrier	5	Yes
	017	Jack Russell Terrier	2	Yes
	018	Jack Russell Terrier	4	Yes
	019	Maltese	3	Yes
3	020	Pomeranian	3	Yes
	021	Shiba Inu	5	Yes
	022	Shetland Sheepdog	2	Yes
	023	Shetland Sheepdog	2	Yes
	024	Shetland Sheepdog	4	Yes
	025	Shetland Sheepdog	4	Yes
	026	Pomeranian	1	No
	027	Pomeranian	4	No
	028	Shiba Inu	5	No
4	029	Cavalier King Charles Spaniel	3	Yes
	030	Miniature Pinscher	2	Yes
	031	Miniature Pinscher	2	Yes
	032	Shih Tzu	1	Yes
	033	Shih Tzu	1	Yes
	034	Shih Tzu	3	Yes
	035	Shih Tzu	3	Yes
	036	Shih Tzu	5	Yes
5	037	Goldendoodle	2	Yes
	038	Goldendoodle	3	Yes

	039	Goldendoodle	4	Yes
	040	Goldendoodle	6	Yes
	041	Miniature Goldendoodle	2	Yes
	042	Miniature Goldendoodle	2	Yes
	043	Miniature Goldendoodle	2	Yes
	044	Miniature Goldendoodle	2	Yes
	045	Goldendoodle	3	No
	046	Goldendoodle	3	No
6	047	Bichon Frise	3	Yes
	048	Cavalier King Charles Spaniel	3	Yes
	049	Cocker Spaniel	4	Yes
	050	Cocker Spaniel	5	Yes
	051	Pomeranian	3	Yes
	052	Miniature Schnauzer	5	Yes
	053	Shiba Inu	3	Yes
	054	Pomeranian	5	No
	055	Pomeranian	4	No
	056	Yorkshire Terrier	5	No
7	057	Australian Shepard	3	Yes
	058	Bichon Frise	3	Yes
	059	Bichon Frise	3	Yes
	060	Havanese	2	Yes
	061	Havanese	2	Yes
	062	Havanese	3	Yes
	063	Miniature Australian Shepard	1	Yes
8	064	Bichon Frise	4	Yes
	065	Bichon Frise	4	Yes
	066	Bichon Frise	5	Yes
	067	Cavalier King Charles Spaniel	1	Yes
	068	Cocker Spaniel	1	Yes
	069	Havanese	2	Yes
	070	Havanese	3	Yes
	071	Havanese	3	Yes
	072	Havanese	4	Yes
	073	Cavalier King Charles Spaniel	2	No
	074	Havanese	3	No

*Subjects not included in the analysis were dropped due to various reasons such as lack of pregnancy, no living puppies, or whelping dates causing travel conflicts.

Table S2. Linear mixed-effects model output for fixed effects. Statistically significant *p*-values are bolded.

**Metric**	**Estimate**	**SE**	**df**	**t-value**	***p*-value**

FIDO+ (All)	(Intercept)	3.58	0.43	147	8.24	-
	Time: 4 weeks postpartum	0.58	0.30	147	1.95	**0.05**
	Time: 1 week prepartum	-0.07	0.34	147	-0.20	0.84
	Time: 6 weeks postpartum	-0.22	0.29	147	-0.75	0.45
FIDO+ (Treats)	(Intercept)	0.89	0.16	147	5.59	0
	Time: 4 weeks postpartum	0.41	0.12	147	3.40	**0.0009**
	Time: 1 week prepartum	0.05	0.14	147	0.36	0.72
	Time: 6 weeks postpartum	0.03	0.12	147	0.24	0.81
HCC	(Intercept)	2.89	0.12	134	24.24	-
	Time: 4 weeks postpartum	-0.27	0.09	134	-3.00	**0.003**
	Time: 1 week prepartum	-0.10	0.09	134	-1.13	0.26
	Time: 6 weeks postpartum	-0.13	0.08	134	-1.57	0.12
FGM	(Intercept)	5.87	0.16	114	36.03	-
	Time: 4 weeks postpartum	-0.08	0.15	114	-0.59	0.56
	Time: 1 week prepartum	-0.46	0.17	114	-2.72	**0.008**
	Time: 6 weeks postpartum	-0.04	0.17	114	-0.22	0.83
FIDO+ (Behavior)	(Intercept)	2.69	0.32	147	8.53	-
	Time: 4 weeks postpartum	0.18	0.22	147	0.80	0.43
	Time: 1 week prepartum	-0.12	0.26	147	-0.46	0.65
	Time: 6 weeks postpartum	-0.25	0.22	147	-1.13	0.26
Fecal sIgA	(Intercept)	1.30	0.19	113	6.72	-
	Time: 4 weeks postpartum	-0.53	0.23	113	-2.32	**0.02**
	Time: 1 week prepartum	-0.43	0.26	113	-1.70	0.10
	Time: 6 weeks postpartum	-0.50	0.26	113	-1.91	0.06

Table S3. Pairwise comparison output. Statistically significant *p*-values are bolded.

Metric	Contrast	Estimate	SE	t-ratio	*p*-value
FIDO+ (All)	8 to 4 weeks postpartum	-0.58	0.30	-1.95	0.21
	8 weeks postpartum to 1 week postpartum	0.07	0.34	0.20	1.0
	8 weeks postpartum to 6 weeks prepartum	0.22	0.29	0.76	0.87
	4 weeks postpartum to 1 week prepartum	0.65	0.34	1.91	0.23
	4 weeks postpartum to 6 weeks prepartum	0.80	0.29	2.76	**0.03**
	1 to 6 weeks prepartum	0.15	0.34	0.46	0.97
FIDO+ (Treats)	8 to 4 weeks postpartum	-0.41	0.12	-3.40	**0.005**
	8 weeks postpartum to 1 week postpartum	-0.05	0.14	-0.36	0.99
	8 weeks postpartum to 6 weeks prepartum	-0.03	0.12	-0.24	1.0
	4 weeks postpartum to 1 week prepartum	0.36	0.14	2.62	**0.05**

	4 weeks postpartum to 6 weeks prepartum	0.38	0.12	3.24	**0.008**
	1 to 6 weeks prepartum	0.02	0.14	0.15	1.0
HCC	8 to 4 weeks postpartum	0.27	0.09	3.00	**0.02**
	8 weeks postpartum to 1 week prepartum	0.10	0.09	1.13	0.67
	8 weeks postpartum to 6 weeks prepartum	0.13	0.08	1.57	0.40
	4 weeks postpartum to 1 week prepartum	-0.17	0.09	-1.80	0.28
	4 weeks postpartum to 6 weeks prepartum	-0.14	0.09	-1.59	0.39
	1 to 6 weeks prepartum	0.03	0.09	0.031	0.99
FGM	8 to 4 weeks postpartum	0.09	0.15	0.59	0.94
	8 weeks postpartum to 1 week prepartum	0.46	0.17	2.72	**0.04**
	8 weeks postpartum to 6 weeks prepartum	0.04	0.17	0.22	1.0
	4 weeks postpartum to 1 week prepartum	0.37	0.17	2.19	0.13
	4 weeks postpartum to 6 weeks prepartum	-0.05	0.17	-0.29	0.99
	1 to 6 weeks prepartum	-0.42	0.19	-2.24	0.12
